# Association between Paclitaxel Clearance and Tumor Response in Patients with Esophageal Cancer

**DOI:** 10.3390/cancers11020173

**Published:** 2019-02-01

**Authors:** Eelke L.A. Toxopeus, Femke M. de Man, Nanda Krak, Katharina Biermann, Annemieke J.M. Nieuweboer, Lena E. Friberg, Esther Oomen-de Hoop, Jan J.B. van Lanschot, Joel Shapiro, Bas P.L. Wijnhoven, Ron H.J. Mathijssen

**Affiliations:** 1 Department of Surgery, Erasmus University Medical Center, Doctor Molewaterplein 40, 3015 GD Rotterdam, The Netherlands; e.toxopeus@erasmusmc.nl (E.L.A.T.); j.vanlanschot@erasmusmc.nl (J.J.B.v.L.); shapirox@gmail.com (J.S.); b.wijnhoven@erasmusmc.nl (B.P.L.W.); 2 Department of Medical Oncology, Erasmus Medical Center Cancer Institute, Erasmus University Medical Center, Doctor Molewaterplein 40, 3015 GD Rotterdam, The Netherlands; f.deman@erasmusmc.nl (F.M.d.M.); nieuweboer.ajm@gmail.com (A.J.M.N.); e.oomen-dehoop@erasmusmc.nl (E.O.-d.H.); 3 Department of Radiology, Erasmus University Medical Center, Doctor Molewaterplein 40, 3015 GD Rotterdam, The Netherlands; nc_krak@hotmail.com; 4 Department of Pathology, Erasmus University Medical Center, Doctor Molewaterplein 40, 3015 GD Rotterdam, The Netherlands; k.biermann@erasmusmc.nl; 5 Department of Pharmaceutical Biosciences, Uppsala University, Box 256, 751 05 Uppsala, Sweden; lena.friberg@farmbio.uu.se

**Keywords:** paclitaxel, esophageal cancer, treatment response, pharmacokinetics

## Abstract

Inter-individual variability in paclitaxel pharmacokinetics may play a role in the response to chemotherapy. Therefore, we studied the association between paclitaxel clearance and treatment response in patients with esophageal cancer. All patients who received paclitaxel (plus carboplatin) treatment for esophageal cancer between 2007 and 2013 were included. The treatment was given as neoadjuvant chemoradiotherapy (nCRT), induction chemotherapy (iCT), or palliative chemotherapy (pCT). The treatment response was assessed by the tumor regression grade (TRG) or by the RECIST1.1 criteria, respectively. The unbound paclitaxel clearance (CL) was estimated with NONMEM. The log-transformed clearance was related to response with ANOVA and independent sample t-tests. A total of 166 patients were included, of whom 113 received nCRT, 23 iCT and 30 pCT. In patients receiving nCRT, paclitaxel clearance was not associated with tumor regression grade (*p*-value = 0.25), nor with pathologically complete response (geometric mean 561.6 L/h) and residual disease (geometric mean 566.1 L/h, *p*-value = 0.90). In patients who underwent iCT or pCT, also no association between paclitaxel clearance and RECIST outcome was identified (iCT: *p*-value = 0.08 and pCT: *p*-value = 0.81, respectively). In conclusion, systemic paclitaxel exposure was not associated with response to common paclitaxel-based treatment regimens for esophageal cancer. Future studies should focus on tumor exposure in relation to systemic exposure and treatment outcome.

## 1. Introduction

The incidence of esophageal cancer is still rising in the United States and Western Europe and mortality is high [1,2]. Esophageal cancer is often diagnosed at an advanced stage. Therefore, curative treatment is only attempted in less than fifty percent of patients [3]. Based on the evidence from the Dutch randomized CROSS trial, paclitaxel can be used in combination with carboplatin and radiotherapy as an effective neoadjuvant treatment strategy [4,5]. In approximately 30% of patients, no vital tumor cells are left in the esophagectomy specimen following neoadjuvant chemoradiotherapy (nCRT) [4,6,7]. In another 30% of patients, partial regression of the tumor is observed (1–10% vital tumor cells), while in 25% of patients the resection specimen does not show changes in regression (>50% of vital tumor cells). In patients with extensive disease not amenable for surgery, induction or palliative chemotherapy (iCT or pCT, respectively) is given, where paclitaxel is also combined with carboplatin [8,9,10,11]. In this setting, the dose of paclitaxel is higher (weekly 100 mg/m^2^) than in the neoadjuvant setting (weekly 50 mg/m^2^).

Paclitaxel is a classic chemotherapeutic agent which stabilizes cellular microtubules, thereby blocking chromosomal segregation and mitosis, and eventually inducing apoptosis [12,13]. There is a suggested dose-response relationship for this agent [14,15]. Unfortunately, paclitaxel is also known for its huge inter-individual variability in pharmacokinetics, which is largely explained by (pharmaco-) genetic and environmental differences between patients [14]. As a consequence, differences in (dose-limiting) toxicities may be explained by differences in systemic exposure between patients [14,16,17]. However, if differences in outcome could also be explained by the variation in systemic paclitaxel exposure, is currently unknown. 

Therefore, we hypothesized that an increased systemic paclitaxel exposure (due to low clearance) is associated with a better response to treatment for patients with esophageal cancer. Therefore, in this study, for the first time, the association between systemic exposure to paclitaxel and therapeutic effect in patients with esophageal cancer was studied. 

## 2. Results

A total of 166 patients with esophageal cancer were included from a prospectively collected database, of whom 113 patients underwent neoadjuvant chemoradiotherapy followed by surgery. Another 23 patients received induction chemotherapy (of whom 11 proceeded to esophagectomy) and 30 patients underwent palliative treatment. Patient and tumor characteristics of all enrolled patients are listed in Table 1. 

The majority of the patients were male and had an esophageal adenocarcinoma. In patients receiving neoadjuvant chemoradiotherapy, as well as induction and palliative chemotherapy: cT3 status, cN1 status, a moderately differentiated tumor, and located at the distal esophagus was seen most. Not all patients received the initially planned courses due to toxicity or the patient’s condition (Table 1).

The results for individual paclitaxel clearance as the measure for paclitaxel exposure is listed per treatment and response group in Table 2. The paclitaxel clearance is expressed as the geometric mean (GM) with the coefficient of variation (CV).

Thirty-six patients who underwent neoadjuvant CRT had a pathologically complete response (32%) and 77 patients (68%) had a partial or no response, based on their esophagectomy specimen. The tumor regression grade was not significantly associated with paclitaxel clearance (*p*-value = 0.25, Table 2). Post-hoc tests were not performed because of the non-significant overall effect. Also, when comparing the clearance of patients with a pathologically complete response (TRG1) to the clearance of patients with residual disease (TRG 2–4) no difference was seen (geometric mean ratio = 0.99, 95% CI [0.87–1.13], *p*-value = 0.90, Table 3). 

The radiological classification of patients—treated either by induction or by palliative chemotherapy—is also listed in Table 2. In none of the 23 patients who underwent induction chemotherapy, a progression of disease was seen. A complete response was seen in two patients, partial response in 12 patients and stable disease in nine patients. The response grade according to the modified RECIST1.1 was not statistically significantly associated with response (*p*-value = 0.08, Table 2). However, a possible trend was seen towards a better response in patients with an increasing paclitaxel exposure, although the number of patients with a clinical complete response was only two. Some 30 patients treated with palliative intent were evaluated in the current analysis of whom eight patients (27%) had a progression of the disease at a moment of response evaluation after 6 cycles of chemotherapy. Also in this group, we could not identify an association between paclitaxel clearance and tumor response (*p*-value = 0.81, Table 2). 

## 3. Discussion

To our knowledge, this is the first study that investigated the association between systemic exposure to paclitaxel and tumor response in patients with esophageal cancer. Response to paclitaxel in patients receiving neoadjuvant chemoradiotherapy (nCRT), induction chemotherapy or palliative chemotherapy was analyzed. In contrast to what was hypothesized, systemic concentrations of paclitaxel were not associated with pathological response or radiological tumor regression. 

In patients receiving induction chemotherapy, a possible trend was seen towards patients with a clinical complete response having a lower paclitaxel clearance than patients with a partial response or stable disease. However, as only two patients had a clinically complete response in this subgroup, no hard conclusions can be drawn on this point. 

One of the potential reasons why a relationship between pharmacokinetics and response was not seen could be that in patients receiving neoadjuvant chemoradiotherapy, the chemotherapy mainly acted as a radiosensitizer [18,19,20]. Thus, the effects of paclitaxel exposure on treatment outcome could be overshadowed by the combination with radiotherapy. In addition, the combination with carboplatin chemotherapy (of which no drug concentrations were measured) could have influenced the outcomes of the analyses. Furthermore, the type of tumor (adenocarcinoma versus squamous cell carcinoma) affects the response to chemoradiotherapy. Squamous cell carcinoma reacts more effectively to chemoradiotherapy, as indicated by the fact that a pathological response occurs more often in patients with squamous cell carcinomas. However, the CROSS regimen does not distinguish between the two tumor types in clinical practice [4,5,21]. In the present study, the majority of patients were diagnosed with adenocarcinoma of the esophagus, in line with the incidence in the Western world [22]. 

Another important reason for the lack of correlation between paclitaxel plasma pharmacokinetics and tumor response was the potential weak correlation between paclitaxel plasma exposure and paclitaxel tumor exposure. As one of its potential resistance mechanisms, a tumor may use efflux transporters (i.e. ATP-binding cassette (ABC) transporters) to limit intra-tumoral chemotherapy concentrations. Taxanes, including paclitaxel, are known substrates for these transporters [23,24]. Although we did not measure intra-tumoral drug concentrations in this study, due to its retrospective nature, we speculate that intra-tumoral paclitaxel concentrations differed substantially from plasma chemotherapy concentrations. To further explore this, we recently set up a new prospective clinical trial (the PAREO study; registered at www.trialregister.nl as NTR6356, study number MEC 16.696) in which plasma paclitaxel exposure is compared with intra-tumoral concentrations by serial tumor biopsies and simultaneous blood sampling in patients treated for esophageal cancer. 

Our study has several limitations. The limited sample size of the induction and palliative treatment group could have influenced our results. However, we do think that a strong relationship between paclitaxel clearance and response still could have been detected. Nevertheless, the results of these two treatment groups should be interpreted with caution. Furthermore, not all blood samples were collected during the first treatment cycle resulting in different paclitaxel dosages, especially in the induction and palliative treatment group. However, we used clearance as a measure for systemic exposure, which will not be strongly influenced by drug dosage. Next to this, most patients receiving palliative chemotherapy were treated with six cycles, while others received more. The response evaluation was performed after six weekly cycles (for the first time) in every patient, but the obtained blood samples for clearance were not strictly regulated to these first six weeks. This feature can be of clinical influence on the response, but the numbers were too small to characterize. 

In summary, in this study, the association between systemic exposure to paclitaxel and pathological response/clinical outcome in patients with esophageal cancer was studied. The current analysis demonstrated that systemic paclitaxel exposure was not related to response to common paclitaxel-based treatment regimens for esophageal cancer. Future studies should therefore, focus on intra-tumoral exposure in relation to systemic exposure and treatment outcome.

## 4. Materials and Methods

### 4.1. Patients

All patients were treated at the Erasmus MC Cancer Institute, Rotterdam, the Netherlands, which is a tertiary referral center for patients with esophageal cancer. Patients, aged 18 years or older, treated with paclitaxel for histologically proven carcinoma of the intrathoracic esophagus or gastro-esophageal junction between November 2007 and May 2013, were identified from an institutional database (based on a prospective trial registered at www.trialregister.nl as NTR2311, study number MEC 03.264). In this study, all patients who received paclitaxel mono- or combination-therapy, were included. For pharmacokinetic purposes, a limited sampling strategy was used. All patients with esophageal cancer received either paclitaxel in a neoadjuvant chemoradiotherapy regimen, as induction treatment or in a palliative setting. For each individual patient, a treatment plan was conducted and evaluated during a weekly multidisciplinary team meeting. The ethical approval was given by the ethical committee of the Erasmus MC as an amendment to the prospective trial (NTR2311). All patients provided written informed consent for the mentioned trial.

### 4.2. Staging

The tumors were (re-)staged according to the 7^th^ UICC-AJCC TNM staging manual [25]. Every patient underwent physical examination and routine biochemical and hematological tests. In every patient, an upper gastrointestinal endoscopy with biopsies, computed tomography (CT) of the neck, chest and abdomen, and external ultrasonography of the neck with Fine-Needle Aspiration (FNA) in case of suspected lymph nodes, was performed according to the Dutch esophageal cancer guidelines. Only in T3 tumors, was Positron Emission Tomography (PET) proven to be of any additional value at that time, and was not yet standardized.

### 4.3. Neoadjuvant Chemoradiotherapy

On days 1, 8, 15, 22, and 29, paclitaxel and carboplatin were administered intravenously. A paclitaxel dose of 50 mg/m^2^ was administered and the targeted area under the curve (AUC) was 2 mg/mL/min for carboplatin. A total 3D conformal radiation dose of 41.4 Gy was given in 23 fractions of 1.8 Gy each, with 5 fractions administered per week. Radiotherapy started on the first day of the first chemotherapy cycle [4,21].

### 4.4. Induction or Palliative Chemotherapy

Weekly 100 mg/m^2^ paclitaxel was given together with carboplatin targeting at an AUC of 4 mg/mL/min [26,27]. In some patients, induction or palliative chemotherapy was continued beyond the planned number of six cycles. This was done to either sustain tumor regression, or in case of partial response, for further downsizing tumor volume. The regimen these patients received consisted of 175 mg/m^2^ paclitaxel and carboplatin (targeted at an AUC of 6 mg/mL/min) and administered in three 3-weekly cycles.

### 4.5. Surgery

If surgery was feasible (after neoadjuvant chemoradiotherapy or after successful induction chemotherapy), operations were performed or strictly supervised by experienced upper-GI surgeons in four hospitals, specialized in esophageal surgery. For tumors of the intrathoracic esophagus and for junctional tumors with positive lymph nodes at or above the carina, a transthoracic approach with two-field lymph node dissection was generally performed. In patients with a poor performance status (WHO performance score of 2 or higher) or for tumors substantially involving the gastro-esophageal junction, a transhiatal resection was favored [28,29]. 

### 4.6. Response Evaluation

In patients treated with neoadjuvant chemoradiotherapy, the treatment response was based on the assessment of the resection specimen. After surgery, the resection specimens were immediately sent to the Department of Pathology and instantly examined by the attending pathologist. The samples of the tumor, lymph nodes and resection margins were obtained before the specimen was fixed in formalin. A radical resection (ypR0, where yp means pathological after neoadjuvant treatment) was defined as no tumor cells within 1 mm of the circumferential, proximal or distal resection margins [4]. Hence, when tumor cells were detected at or within 1 mm of the resection plane it was classified as ypR1. The number of lymph nodes removed and the number of tumor positive lymph nodes removed were assessed. The tumor regression grade (TRG), used to assess the response to neoadjuvant chemoradiotherapy or to induction chemotherapy, was classified into four categories according to a modified Mandard score. TRG 1 means there were no vital tumor cells in the resection specimen (pathologically complete response of the primary tumor and removed lymph nodes, ypT0N0M0); TRG 2 means there were less than 10% residual vital tumor cells and/or any residual vital tumor cells in the lymph nodes; TRG 3 means there were between 10 and 50% residual vital tumor cells; and TRG 4 means there were more than 50% residual vital tumor cells [6,30]. For this study, all samples were re-analyzed by one pathologist (K.B.).

In patients treated with induction or palliative chemotherapy, the treatment response was assessed using CT images after six weekly cycles of chemotherapy and scored according to the “response evaluation criteria in solid tumors” (RECIST) classification system [31]. A modified RECIST 1.1. score was used, where smaller lesions than required according to definitions for RECIST 1.1 were taken into account as well. All CT images were re-evaluated by a single radiologist (N.K.). If no tumor lesions were seen on the CT imaging after induction or palliative chemotherapy, the patients were classified as having a complete response (CR). When imaging showed regression of the primary tumor and/or lymph nodes or the presence of novel metastatic lesions, the patients were classified as having a partial response (PR). If there was no difference in tumor and/or lymph node size and metastatic lesions, the patients were classified as having stable disease (SD). In case of progression in size of the primary tumor and/or lymph nodes or metastatic lesions or development of new lesions, the patients were classified as having progressive disease (PD) [31]. 

### 4.7. Paclitaxel Pharmacokinetic Analyses

The analyses for paclitaxel pharmacokinetics were performed according to previous studies [14,16,17]. In brief, from each patient, blood was taken during one of the five or six (dependent of the type of treatment) weekly chemotherapy cycles, using a formerly endorsed limited sampling strategy with 4 to 5 samples within approximately 24 hours after the start of paclitaxel infusion [14,32]. To prevent coagulation, lithium heparin was used in all samples. Subsequent to sample collection, paclitaxel concentrations were determined using a validated method [16]. Next to individual total paclitaxel plasma concentrations, a well-established population pharmacokinetic model and NONMEM software (Icon Development Solutions, Leopardstown Dublin, Dublin, Ireland) were used to determine the paclitaxel clearance (CL, L/h) in each individual patient [14]. 

### 4.8. Statistical Analysis

The primary outcome of this study was the association between paclitaxel clearance and response to systemic treatment in patients with esophageal cancer. The analyses of the unbound paclitaxel clearance were performed on log-transformed clearance values, since they were assumed to follow a log-normal distribution. Hence, clearance was described by means of geometric means and corresponding coefficients of variation (CV). The differences in clearance between TRG groups were tested by means of ANOVA. The post-hoc tests were only performed if the overall (omnibus) test was significant at the 5% level without correction for multiple testing. The difference between patients with a complete response (TRG1) and patients with residual disease (TRG2-4) was tested by means of the independent samples t-test. In order to interpret the difference found on the log-scale, the difference and corresponding 95% confidence interval (CI) boundaries were exponentiated to represent the geometric mean ratio and its CI on the original scale. The statistical analyses were performed with the use of SPSS software, version 22.0 (SPSS, IBM, New York, NY, USA). 

## 5. Conclusions

This is the first study, which evaluated the relation of individual paclitaxel plasma pharmacokinetics and treatment response in patients with esophageal cancer treated with a regimen of chemoradiotherapy, including paclitaxel. An association between paclitaxel pharmacokinetics and response could not be demonstrated. The challenge to predict response to treatment remains highly relevant to come to true personalized medicine. 

## Figures and Tables

**Table 1:** Patient and tumor characteristics.

Characteristic	nCRT (n = 113)	iCT (n = 23)	pCT (n = 30)
Sex, n (%)			
Male	91 (80.5%)	16 (69.6%)	29 (96.7%)
Age, years (median and range)	63 (39–82)	64 (52–77)	64 (47–76)
Tumor type			
Adenocarcinoma	90 (79.6%)	13 (56.5%)	24 (80.0%)
Squamous cell carcinoma	22 (19.5%)	9 (39.1%)	6 (20.0%)
Other +	1 (0.9%)	1 (4.3%)	
Histopathological grading			
G1	3 (2.5%)	0	2 (6.7%)
G2	51 (45.1%)	7 (30.4%)	5 (16.7%)
G3	32 (28.3%)	10 (43.5%)	13 (43.3%)
G4	1 (0.9%)	0	0
Gx or Missing	26 (23.0%)	6 (26.1%)	10 (33.3%)
Tumor localization			
Proximal	0	2 (8.7%)	0
Middle	18 (15.9%)	5 (21.7%)	4 (13.3%)
Distal	80 (70.8%)	10 (43.5%)	19 (63.3%)
Gastro-esophageal junction	15 (13.3%)	6 (26.1%)	7 (23.3%)
Clinical T stage			
cT1	4 (3.5%) *	0	0
cT2	26 (23.0%) *	0	2 (6.7%)
cT3	80 (70.8%) *	17 (73.9%)	16 (53.3%)
cT4	3 (2.7%) *	5 (21.7%)	3 (10.0%)
Missing	0	1 (4.3%)	9 (30.0%)
Clinical N stage			
N0	35 (31.0%) #	3 (13.0%)	3 (10.0%)
N1	41 (36.3%) #	5 (21.7%)	6 (20.0%)
N2	34 (30.1%) #	11 (47.8%)	10 (33.3%)
N3	3 (2.7%) #	4 (17.4%)	5 (16.7%)
Missing	0	0	6 (20.0%)
Clinical M stage			
M0	113 (100%)	21 (91.3%)	2 (6.7%)
M1	0	2 (8.7%) ^	28 (93.3%)
TREATMENT REGIMEN			
Neoadjuvant chemoradiotherapy	113 (100%)	X	X
4 courses of Paclitaxel	3 (2.7%)	X	X
5 courses of Paclitaxel	109 (96.5%)	X	X
6 courses of Paclitaxel	1 (0.9%)	X	X
Induction or palliative chemotherapy	X	23 (100%)	30 (100%)
6 courses of Paclitaxel	X	8 (34.8%)	13 (43.3%)
6 + 3 courses of Paclitaxel	X	15 (65.2%)	17 (56.7%)
Resection	113 (100%)	11 (47.8%)	X
Other treatment	X	X	1 (3.3%) £

Abbreviations: nCRT: neoadjuvant chemoradiotherapy, iCT: induction chemotherapy, pCT: palliative chemotherapy. + Other: neuroendocrine tumor. * uTstage (endosonography) in patients treated with neoadjuvant chemoradiotherapy. # uNstage (endosonography) in patients treated with neoadjuvant chemoradiotherapy. $ no location possible due to only radiological diagnostics. ^ Submucosal metastasis and suspicion of lung metastasis. £ brachytherapy.

**Table 2:** Paclitaxel clearance per treatment and response group.

	Clearance (L/h) (Geometric Mean (CV, %))	*p*-Value
**nCRT (n = 113)**		0.25
TRG1 (n = 36)	561.6 (34)	
TRG2 (n = 28)	591.4 (20)	
TRG3 (n = 37)	578.5 (29)	
TRG4 (n = 12)	478.5 (56)	
**iCT (n = 23)**		0.08
CR (n = 2)	358.1 (37)	
PR (n = 12)	409.9 (29)	
SD (n = 9)	500.7 (8)	
PD (n = 0)	X	
**pCT (n = 30)**		0.81
CR (n = 2)	488.0 (16)	
PR (n = 11)	447.1 (35)	
SD (n = 9)	440.5 (33)	
PD (n = 8)	500.2 (23)	

Abbreviations: nCRT = neoadjuvant chemoradiotherapy; iCT = induction chemotherapy; pCT = palliative chemotherapy; CV = coefficient of variation; TRG = tumor regression grade; CR = complete response, PR = partial response, SD = stable disease, PD = progressive disease.

**Table 3:** Paclitaxel clearance of patients with pathologically complete response versus patients with residual disease after neoadjuvant chemoradiotherapy followed by surgery.

	Clearance (L/h) (Geometric Mean (CV, %))	*p*-Value	Geometric Mean Ratio
**nCRT (n = 113)**		0.90	0.99 (CI [0.87–1.13])
TRG 1 (n = 36)	561.6 (34)		
TRG 2-4 (n = 77)	566.1 (32)		

Abbreviations: nCRT = neoadjuvant chemoradiotherapy; CV = coefficient of variation; TRG = tumor regression grade.
